# Oral supplementation of alkaline phosphatase in poultry and swine

**DOI:** 10.1093/tas/txac079

**Published:** 2022-06-15

**Authors:** Jeffery Escobar, Merilyn Dobbs, Claudia Ellenberger, Alysia Parker, Juan D Latorre, Leslie Gabor

**Affiliations:** Elanco Animal Health, Greenfield, IN 46140, USA; Elanco Australasia Pty. Ltd., Kemps Creek, NSW 2178, Australia; Elanco Australasia Pty. Ltd., Kemps Creek, NSW 2178, Australia; Elanco Australasia Pty. Ltd., Kemps Creek, NSW 2178, Australia; Elanco Animal Health, Greenfield, IN 46140, USA; Elanco Australasia Pty. Ltd., Kemps Creek, NSW 2178, Australia

**Keywords:** alkaline phosphatase, gut health, intestinal permeability, lipopolysaccharide, skin pigmentation, tolerance

## Abstract

The importance of intestinal alkaline phosphatase (IAP) in maintaining gut health and intestinal homeostasis is well established. The objective of this study was to investigate the tolerance of poultry and swine to dietary supplementation of a novel microbial-derived alkaline phosphatase (AP; E.C. 3.1.3.1 produced by *Paenibacillus lentus* strain CMG3709). Studies were conducted on day-old Ross 308 chicken (*n* = 1,000; Study 1) and weaned piglets (*n* = 180; Study 2) for a duration of 42 d; and consisted of four treatment groups (TG) based on the concentration of microbial-derived AP supplemented in their diet at 0; 12,000; 20,000; and 200,000 U/kg of feed. Parameters such as animal survival, hematology, coagulation, and biochemical indices were assessed at the end of the study. The effect of microbial AP on nutrient absorption through skin pigmentation and intestinal permeability were also investigated in broilers (*n* = 600; Study 3). In poultry (Study 1), there were no statistically significant differences between control and TG for any of the hematological and biochemical parameters, except for a marginal increase (*P* < 0.05) in serum phosphorus at the highest dose. This variation was not dose-dependent, was well within the reference range, and was not associated with any clinical correlates. In swine (Study 2), hematological parameters such as leukocyte, basophil, and lymphocyte counts were lower (*P* < 0.05) for the two highest doses but were traced back to individual variations within the group. The biochemical indices in piglets showed no significant differences between control and supplemental groups except for glucose (*P* = 0.0005), which showed a high effect (*P* = 0.008) of the random blood collection order. Nonetheless, glucose was within the normal reference range, and were not related to in-feed supplementation of AP as they had no biological significance. The survival rate in all three studies was over 98%. Dietary supplementation of microbial-derived AP up to 16.7 times the intended use (12,000 U/kg feed) level had no negative effects in both poultry and swine. In-feed supplementation of microbial-derived AP for 28 d improved intestinal pigment absorption (*P* < 0.0001) and reduced intestinal paracellular permeability (*P* = 0.0001) in broilers (Study 3). Based on these results, it can be concluded that oral supplementation of microbial-derived AP is safe for poultry and swine and effective at improving gut health in poultry.

## INTRODUCTION

Gut health plays a pivotal role in the performance of farm animals and is of significant interest to the animal science research community. There is accumulating scientific evidence on the benefits of intestinal alkaline phosphatase (IAP) in maintaining gut health in monogastric animals (Lallès, 2010; Melo et al., 2016). IAP is an isoform of alkaline phosphatase (AP, Enzyme Commission 3.1.3.1), which is a family of enzymes that hydrolyze organic phosphate monoesters. Isoforms of mammalian AP are classified into two groups the tissue nonspecific alkaline phosphatases (TNAP), predominantly expressed in liver, kidney, and bones and the other are tissue-specific AP, germ cell alkaline phosphatase (GCALP), placental alkaline phosphatase (PLAP) expressed in thymus and testis, and IAP (Estaki et al., 2014). IAP, is a brush border enzyme, expressed and secreted by enterocytes, it is active within the mucosal membrane and is secreted into the intestinal lumen, serum, and in stool (Genova et al., 2020).

The gut serves as a selective permeable barrier to aid in the movement of nutrients to the inside of the body and is the most vulnerable site for infection. The gut immune system experiences constant challenges to keep an optimal balance between the healthy commensal bacteria and the pathogenic microbes. Hence, the gut is equipped with a strong mucosal immunity, consisting of gut-associated lymphoid tissue (GALT), lamina propria, and epithelial cells, which form a protective barrier to maintain the integrity of the intestinal tract. IAP is an integral part of gut mucosal defense (Goldberg et al., 2008). IAP is known to dephosphorylate bacterial lipopolysaccharide (LPS) which is predominantly produced by Gram-negative bacteria, leading to reduction of inflammation in the gut (Lee et al., 2014; Genova et al., 2020), regulate protective surface microclimate by dephosphorylation of luminal ATP and modulating intestinal pH (Akiba et al., 2007; Mizumori et al., 2009), maintain inorganic phosphate homeostasis (Sasaki et al., 2018), dephosphorylate uridine diphosphate which is involved in inflammatory cascade (Moss et al., 2013), and reduce bacterial translocation (Wang et al., 2015). Overall, IAP plays an integral role in maintaining intestinal homeostasis. Several studies in animals have established that oral supplementation of IAP in feed can assist in the amelioration of intestinal and systemic inflammation in a variety of diseases including dysbiosis and inflammatory bowel disease (Genova et al., 2020). Gut-associated diseases are easily transmitted and have a high morbidity rate, leading to poor feed conversion ratios causing large economic losses in production animals. Administration of IAP as a feed additive has the potential to improve gut health and performance in poultry and swine production.

The aim of our studies was to determine the tolerance of supplementing a novel microbial-derived AP in the feed of target animal species, poultry and swine. Tolerance was assessed through analysis of hematology, coagulation, and blood biochemical indices. The effect of microbial-derived AP ingestion on intestinal permeability and skin pigmentation was also investigated.

## MATERIALS AND METHODS

### Animal Ethics Committee Approval

The use of animals and all animal procedures were approved by the Elanco Animal Ethics Committee. The studies were conducted under the Australian Pesticide and Veterinary Medicines Authority Small-Scale Trials Permit PER7250.

### Location of the Study Area

The studies were conducted at Yarrandoo Research and Development Centre, Elanco Australasia Pty Ltd. Kemps Creek, NSW, Australia.

### Experimental Animals and Their Management

Clinically healthy day-old male Ross 308 broiler chickens (Gallus gallus domesticus) were sourced for studies 1 and 3 from a commercial hatchery (Aviagen, Goulburn, NSW Australia) vaccinated against Newcastle Disease and Infectious Bronchitis. Unless otherwise indicated, animals had ad libitum access to mash feed and water via nipple drinkers; water was supplied by the local water authority. Animals and feeders were weighed at the beginning and end of each feeding phase.

In Study 1, a total of 1,000 birds were randomly allocated on day 0 at 25 birds in each of 40 floor pens (1.06 m × 2.40 m) located in a climate-controlled facility during the entire study. Artificial lighting was provided according to breeder recommendations and was controlled by automatic timers. Birds were fed starter (0 to 14 d), grower (14 to 28 d), and finisher (28 to 42 d) diets.

In Study 2, a total of 180 piglets (Sus scrofa) from a single lot of weaned piglets were included in the study, with an average age of 25 d and an average body weight (BW) of 7.2 kg. One-half of the selected animals (90 piglets) were intact males and one-half (90 piglets) were females. All piglets were sourced from a commercial piggery (PIC, Grong Grong, NSW, Australia) and underwent a general health check prior to arrival at the research facility. Piglets were randomized to pens, six piglets per pen in 30 pens of 1.4 m × 2.8 m each, which provided more than the required space allowance for pigs up to 30 kg of weight (COE, 2006). The weather-protected animal housing facility allowed natural airflow and indirect light. The piglets were fed appropriate feed in two phases: phase 1 (0 to 21 d) and phase 2 (21 to 42 d). The age and sex of the animals selected for the study were based on the recommendations regarding the design and conduct of target animal safety (TAS) studies (VICH, 2008).

In Study 3, a total of 600 birds were randomly allocated on day 0 at 30 birds in each of 20 floor pens. Pens and facility are described in Study 1. Birds were fed starter (0 to 14 d) and grower (14 to 30 d) diets. Animals in all studies were weighed at the beginning and end of each study.

### Experimental Design

Studies 1 and 2 were designed based on the guidelines regarding the number of animals per treatment and experimental conditions for tolerance studies in target animals (EFSA, 2017). Studies 1 and 2 were conducted as a randomized block design of four microbial-derived novel AP (Elanco Animal Health, Greenfield, IN, USA) treatment groups (TG). Animals were randomized to TG based on BW with equal numbers of each sex within each group in Study 2. Study 3 was conducted as a randomized design of two AP TG. The TG, doses of AP tested and experimental unit for each study are depicted in Table 1.

**Table 1.** Treatment groups in Studies 1, 2, and 3

Treatment group	Alkaline phosphatase dose, U/kg*	Study 1 total birds, (pens)	Study 2 total piglets, (pens)	Study 3 total birds, (pens)
TG1	0	250 (10)	36 (6)	300 (10)
TG2^†^	12,000	250 (10)	48 (8)	300 (10)
TG3	20,000	250 (10)	48 (8)	- - -
TG4	200,000	250 (10)	48 (8)	- - -

Elanco Animal Health, Greenfield, IN, USA; U: units.

In Study 3, the target dose for TG2 was 10,000 U/kg.

### Experimental Feed Additive and Treatments

The AP gene was identified in *Paenibacillus lentus* through genomic sequences and confirmed by cloning it into *Escherichia coli*. The native protein of AP was molecular evolved to confer higher heat tolerance in *E. coli* and self-cloned back onto *P. lentus* chromosome under a strong native promoter for expression, resulting in production strain CMG3709. The AP from CMG3709 was delivered orally in feed in all studies, as the site of action is the lumen of the gastrointestinal tract. The intended commercial dose of the enzyme is 12,000 U of AP per kg of feed. The dose rates tested in studies 1 and 2 were 0; 12,000; 20,000; and 200,000 U/kg of feed were selected based on the Food and Drug Administration and European Food Safety Authority guidelines for tolerance studies, including at least a control group, the recommended dose, and an overdose group fed with a multifold higher dose of the intended use-level.

### Processing of the Feed

Diets were formulated to meet or exceed nutrient recommendations for animals (Table 2; NRC, 2012; Aviagen, 2019). For each feeding phase, basal diet batches were mixed, and all treatment diets were derived from the basal diet batches. Liquid AP was incorporated into feed diets as per the test dosage. A batch of ground wheat, ground barley, or ground maize of appropriate size was used to flush the mixer between phases. No antibiotics or growth promoters were included in any feed. Diets were manufactured by personnel at The University of Sydney, Feed Milling and Pelleting Facility, Camden, NSW, Australia using a standard feed preparation procedure.

**Table 2.** Percent ingredient composition of basal diets and estimated nutrient content (as-fed basis)

Study days	Study 1			Study 2		Study 3	
	0–14	14–28	28–42	0–21	21–42	0–14	14–30
Ingredient							
Wheat	31.18	36.52	37.69	31.94	44.38	- - -	- - -
Soybean meal	31.13	24.89	17.15	15.00	25.00	37.43	24.94
Sorghum	- - -	- - -	- - -	- - -	- - -	54.46	58.10
Full-fat soybean meal	3.00	3.00	6.00	- - -	- - -	- - -	
Maize, ground	20.00	15.00	15.00	20.00	10.00	- - -	5.00
Dried whey	- - -	- - -	- - -	11.11	- - -	- - -	- - -
Barley, ground	5.00	7.50	10.00	8.06	10.00	- - -	- - -
Canola meal	3.00	5.00	6.00	5.00	5.00	- - -	5.00
Fishmeal	- - -	- - -	- - -	3.00	- - -	- - -	- - -
Plasma protein	- - -	- - -	- - -	3.14	- - -	- - -	- - -
Soybean oil	2.79	4.73	5.15	- - -	1.82	3.34	2.55
L-Lysine HCl	0.27	0.24	0.24	0.32	0.33	0.24	0.23
DL-Methionine	0.31	0.25	0.22	0.10	0.07	0.38	0.32
L-Threonine	0.15	0.11	0.08	0.12	0.14	0.17	0.12
L-Tryptophan	- - -	- - -	- - -	0.02	0.02	- - -	- - -
Monocalcium phosphate	- - -	- - -	- - -	0.74	1.43	- - -	- - -
Dicalcium phosphate	1.34	1.04	0.82	- - -	- - -	2.11	1.89
Limestone	1.07	0.99	0.95	0.98	1.11	0.95	0.83
Salt	0.25	0.30	0.25	0.20	0.30	0.25	0.30
Sodium bicarbonate	0.15	0.07	0.14	0.07	0.20	0.16	0.10
Choline chloride	0.10	0.10	0.05	- - -	- - -	0.25	0.25
Phytase^1^	0.01	0.01	0.01	- - -	- - -	- - -	- - -
Vitamin-mineral premix^2^	0.25	0.25	0.25	0.20	0.20	0.20	0.20
Coccidiostat^3^	- - -	- - -	- - -	- - -	- - -	0.06	0.06
Yellow pigment^4^	- - -	- - -	- - -	- - -	- - -	- - -	0.10
Red pigment^5^	- - -	- - -	- - -	- - -	- - -	- - -	0.01
Calculated nutrients							
Metabolizable energy, kcal/kg	3,018	3,134	3,202	3,300	3,300	3,050	3,061
Crude protein, %	22.8	21.1	19.4	21.0	20.7	22.8	21.1
SID^6^ Lys, %	1.26	1.12	1.01	1.30	1.17	1.26	1.12
SID^6^ Thr, %	0.86	0.76	0.68	0.83	0.76	0.86	0.76
SID^6^ Met, %	0.63	0.55	0.49	0.40	0.35	0.65	0.58
SID^6^ TSAA, %	0.95	0.86	0.79	0.75	0.69	0.95	0.87
SID^6^ Trp, %	0.26	0.24	0.21	0.26	0.24	0.26	0.23
Calcium, %	0.95	0.85	0.78	0.76	0.82	0.95	0.86
STTD^7^ of phosphorus, %	- - -	- - -	- - -	0.38	0.38	- - -	- - -
Available phosphorus, %	0.48	0.43	0.39	- - -	- - -	0.48	0.44
Sodium, %	0.16	0.16	0.16	0.32	0.20	0.16	0.17

Axtra Phy 10,000 TPT 2 (DuPont Australia Ltd, Macquarie Park, NSW, Australia).

Provided per each kg of feed the following nutrients. Study 1: vitamin A, 30,000 U; vitamin D, 12,500 U; vitamin E, 69 U; vitamin K, 7.5 mg; riboflavin, 22.5 mg; niacin, 125 mg; pantothenic acid, 45; biotin, 0.50; vitamin B12, 0.06 mg; folic acid, 5 mg; pyridoxin, 12.5 mg; thiamin, 7.5; Cu, 50 mg; I, 2.5 mg, Fe, 100 mg, Mn, 275 mg; Se 0.30 mg; Zn, 225 mg. Study 2: vitamin A, 8,000 U; vitamin D, 1,500 U; vitamin E, 30 U; vitamin K, 1 mg; riboflavin, 4 mg; niacin, 15 mg; pantothenic acid, 20; biotin, 0.50; vitamin B12, 0.01 mg; pyridoxin, 1.5 mg; thiamin, 1; Cu, 20 mg; I, 0.5 mg, Fe, 60 mg, Mn, 50 mg; Se 0.30 mg; Zn, 120 mg. Study 3: vitamin A, 24,000 U; vitamin D, 10,000 U; vitamin E, 56 U; vitamin K, 6 mg; riboflavin, 18 mg; niacin, 100 mg; pantothenic acid, 36; biotin, 0.40; vitamin B12, 0.05 mg; folic acid, 4 mg; pyridoxin, 10 mg; thiamin, 6; Cu, 40 mg; I, 2 mg, Fe, 80 mg, Mn, 220 mg; Se 0.30 mg; Zn, 180 mg.

Maxiban, 50 mg of narasin per each kg of feed (Elanco Animal Health, Inc., Greenfield, IN, USA).

Colortek yellow (Novus International, Inc., St. Charles, MO, USA).

RED Carophyll (DSM Nutritional Products Ltd., Heerlen, The Netherlands).

Standardized ileal digestible.

Standardized total tract digestible.

### Blood Collection and Analyses

The blood samples were collected aseptically in labeled Vacuette tubes. Blood samples for hematological analysis were collected into ethylenediaminetetraacetic acid (EDTA) tubes, for clinical chemistry analysis in plain serum tubes, and in sodium citrate tubes for analysis of coagulation variables.

In Study 1, blood was collected on day 42 from three randomly selected birds in each pen. Birds were euthanized and blood was collected via cardiac puncture. In Study 2, blood was collected randomly from two piglets in each pen (one male and one female; *n* = 60 in total), for clinical pathology on day 42.

Blood specimens collected into citrate tubes were centrifuged at 4 °C at 2,440 × g for 15 min for plasma separation. Blood specimens collected into plain serum tubes were centrifuged at ambient temperature at 2,000×*g* for 10 min ensuring serum separation from blood cells. Both the serum and plasma specimens were transferred and stored in individually labeled cryo-vials. The samples were used to assess routine hematology, biochemical and coagulation variables at the Pre-Clinical Laboratory, Elanco, Yarrandoo R & D Centre.

### Intestinal Permeability and Skin Pigmentation Assessment

In Study 3, skin pigmentation was assessed on 5 birds per pen (50 birds per TG) on day 14, and day 27 using a Chroma Meter CR-300 (Konica Minolta, Tokyo, Japan), the color profile of lightness (L*), redness (a*), and yellowness (b*) (CIE, 1978). Basal diets were formulated using ingredients known for low carotenoid content (i.e., pigment) to minimize skin pigmentation during the first 14 d of the study (Table 2). Then, supplemental yellow and red pigments were added to the diet to induce skin pigmentation (Table 2). On day 30, a lactose solution was administered via crop gavage to 10 birds per pen (100 birds per TG). Blood was collected via cardiac puncture after euthanasia, from 3 birds per pen at each of the following time points: 0, 1, and 2 h post-gavage. The blood samples were centrifuged for the collection of serum which was analyzed for lactose by liquid chromatography with tandem mass spectrometry (LC-MS/MS) and reported as ng of lactose per mL of serum. Lactose was used as a permeability marker because lactose but not galactose appeared in blood when chickens were fed lactose (Rutter et al., 1953) indicating the inability of birds to hydrolyze this disaccharide. Further, lactase activity is either not present (Hamilton and Mitchell, 1924) in the gastrointestinal tract of chickens or found at very low levels in isolated enterocytes (Chotinsky et al., 2001; Mozdziak et al., 2003).

### Feed Assay

Feed samples were collected and stored at −20 °C until analysis. Samples were grounded in a centrifugal mill (ZM 200, Retsch GmbH, Haan, Germany) fitted with a 1-mm screen, extracted with buffer (10 mM Tris, 0.1 mM MgCl_2_, and 0.1 mM ZnCl_2_, 0.1% BSA at pH 7.5), and assayed in triplicate for alkaline phosphatase using p-nitrophenyl phosphate (Morgenstern et al., 1965) in an autoanalyzer (Cedex Bio HT Analyzer, Roche Diagnostics GmbH, Mannheim, Germany). One unit of AP activity is defined as the conversion of one μmole per minute of p-nitrophenyl phosphate to p-nitrophenol at pH 10.0 and 40 °C.

### Statistical Analysis

Data from Study 1 were subjected to analysis of variance (ANOVA) using Statistix 9.0 (Analytical Software, Tallahassee, FL, USA), and Duncan’s Multiple Range Test. In Study 2, data were subjected to one-way ANOVA using the Fit Model option of JMP (SAS Institute, Cary, NC, USA). In Studies 1 and 2 alkaline phosphatase level (0; 12,000; 20,000; 200,000) was a fixed effect whereas block was a random effect. Multiple mean comparisons were done using a two-sided Tukey test with *α* = 0.05. In Study 3, intestinal permeability variables were analyzed using the Fit Model option of JMP and mean comparisons were done using a one-sided Student’s *t*-test for skin pigmentation and slope-ratio analysis for serum lactose.

## RESULTS AND DISCUSSION

These studies were designed to assess the tolerance level of a novel microbial-derived AP enzyme added to broiler and swine diets, at the intended use level (12,000 U/kg feed), and multifold higher doses (20,000 and 200,000 U/kg feed) than the intended use level, to establish safety and to assess tolerance limit in the target animals (EFSA, 2017). All animals were healthy throughout the study and their performance was satisfactory although the studies were not designed or powered to detect differences in growth performance among TG (Table 3). Overall animal survival rate in all studies was high with no statistically significant differences detected among TG (Table 3). Addition of AP to the feed was well tolerated and found to be safe up to 16.7 times the intended dose in the target animals. As per EFSA guidelines on the safety for the target species recommendation (EFSA, 2017), the physiological effects of inclusion of AP in the feed were assessed based on hematological, biochemical, and coagulation parameters.

**Table 3.** Body weight and survival after 42 d of in-feed supplementation with microbial-derived alkaline phosphatase in broiler chickens (Study 1) and weaned pigs (Study 2), and after 28 d of in-feed supplementation with microbial-derived alkaline phosphatase in broiler chickens (Study 3)

Study	Item	Alkaline Phosphatase, U/kg				SEM	*P*-value
		0	12,000*	20,000	2000,00		
1	Initial BW, kg	0.035	0.035	0.036	0.035	0.003	0.26
	Final BW, kg	2.87	2.90	2.91	2.89	0.04	0.90
	ADFI, g/d	108.7	110.9	112.6	113.6	2.3	0.19
	ADG, g/d	68.3	67.5	68.4	67.9	0.9	0.90
	FCR, g/g	1.594	1.643	1.651	1.675	0.037	0.24
	Survival, %	100.0	99.2	98.8	98.8	0.5	0.27
2	Initial BW, kg	7.3	7.2	7.1	7.1	0.1	0.72
	Final BW, kg	28.7	29.6	29.5	27.9	0.7	0.22
	ADFI, g/d	907	947	953	1,005	45	0.48
	ADG, g/d	509	533	534	495	17	0.22
	FCR, g/g	1.778	1.775	1.814	2.053	0.114	0.29
	Survival, %	97.2	100.0	100.0	97.9	1.6	0.52
3	Initial BW, kg	0.039	0.039	- - -	- - -	0.004	0.67
	Final BW, kg	1.28	1.32	- - -	- - -	0.03	0.17
	ADFI, g/d	85.1	82.7	- - -	- - -	1.8	0.18
	ADG, g/d	44.3	45.6	- - -	- - -	1.0	0.18
	FCR, g/g	1.936	1.809	- - -	- - -	0.071	0.11
	Survival, %	99.0	97.7	- - -	- - -	0.6	0.16

*In Study 3, the target dose was 10,000 U/kg.

### Alkaline Phosphatase in Feed

The average recovery of alkaline phosphatase in U/kg of feed was for Study 1: TG1 83 (0 expected); TG2 13,775 (12,000 expected); TG3 22,806 (20,000 expected); and TG4 236,660 (200,000 expected); for Study 2: TG1 76 (0 expected); TG2 13,312 (12,000 expected); TG3 23,826 (20,000 expected); and TG4 195,983 (200,000 expected); and for Study 3: TG1 < 50 (0 expected); TG2 12,133 (10,000 expected).

### Hematological and Biochemical Indices

Hematological and biochemical indices of Study 1 are presented in Tables 4 and 5. Red blood cell indices of the birds were within published reference ranges (Bounous et al., 2000; Talebi et al., 2005; Abdi-Hachesoo et al., 2011), and there were no statistical differences noticed among TG. Leukocyte/white blood cell (WBC) count is frequently associated with inflammatory response, or, in some cases, as an indicator of infectious processes. (Kokosharov, 1998). In this study, there was no significant statistical difference noticed in leukocyte counts among TG and the values were within published range (Feldman et al., 2000).

**Table 4.** Hematology, differential cell count, and coagulation parameters after 42 d of in-feed supplementation with microbial-derived alkaline phosphatase in broiler chickens: Study 1

Item	Unit	Alkaline phosphatase, U/kg				SEM	*P*-value
		0	12,000	20,000	200,000		
n	- - -	10	10	10	10	- - -	- - -
RBC	×10^12^/L	2.03	2.03	2.09	2.06	0.07	0.94
HB	g/L	80.23	81.80	79.93	77.97	1.99	0.42
HCT	%	26.37	26.40	26.67	25.83	0.43	0.62
MCV	fl	134.67	133.13	131.03	129.37	3.65	0.80
MCH	pg	41.10	41.23	39.10	39.20	1.14	0.50
MCHC	g/L	304.93	310.07	299.73	302.03	4.34	0.32
THR	×10^9^/L	26.40	23.47	21.33	30.33	3.08	0.12
WBC	×10^9^/L	18.08	16.70	18.40	20.67	2.08	0.36
NEUT	×10^9^/L	5.20	5.27	5.74	6.85	0.68	0.23
LYMPH	×10^9^/L	9.80	8.63	9.81	10.17	1.02	0.59
MONO	×10^9^/L	1.51	1.34	1.46	1.84	0.24	0.31
EOS	×10^9^/L	0.60	0.64	0.59	0.85	0.203	0.30
BASO	×10^9^/L	0.99	0.81	0.80	0.94	0.133	0.58
Fib	g/L	0.75	0.71	0.71	0.78	0.03	0.29

BASO, basophils; EOS, eosinophils; Fib, fibrinogen; HB, hemoglobin; HCT, hematocrit; LYMPH, lymphocytes; MCV, mean corpuscular volume; MCH, mean corpuscular hemoglobin; MCHC, mean corpuscular hemoglobin concentration; MONO, monocytes; NEUT, neutrophils; RBC, red blood cells; SEM, standard error of mean; THR, thrombocytes; U, units; WBC, white blood cells.

**Table 5.** Clinical chemistry parameters after 42 d of in-feed supplementation with microbial-derived alkaline phosphatase in broiler chickens: Study 1

Item	Unit	Alkaline Phosphatase, U/kg				SEM	*P*-value
		0	12,000	20,000	200,000		
n	- - -	10	10	10	10	- - -	- - -
AST	U/L	387	399	425	442	21	0.37
ALT	U/L	1.77	1.73	1.80	1.73	0.18	0.99
CK	U/L	22,389	23,428	22,718	27,320	2,712	0.66
ALP	U/L	4,573	4,481	4,306	3,618	329	0.15
Uric acid	µmol/L	425.6	434.3	402.5	413.6	14.7	0.49
TP	g/L	27.5	27.4	27.3	27.7	0.4	0.89
TBIL	µmol/L	1.60	1.78	1.69	1.76	0.11	0.27
ALB	g/L	10.7	10.6	10.6	10.9	0.1	0.65
GLOB	g/L	16.8	16.7	16.7	16.9	0.3	0.98
CHOL	mmol/L	3.35^a,b^	3.21^a,b^	3.13^b^	3.43^a^	0.07	0.03
AMY	U/L	597	521	549	1,636	521	0.37
GLU	mmol/L	14.8	14.7	14.7	14.5	0.2	0.64
P	mmol/L	2.29^b^	2.37^a,b^	2.32^a,b^	2.44^a^	0.03	0.02
Na	mmol/L	150.7	150.9	150.0	151.0	0.6	0.46
Mg	mmol/L	0.940^a,b^	0.960^a,b^	0.907^b^	0.963^a^	0.029	0.03
Cl	mmol/L	113.2	112.2	112.8	113.0	0.4	0.26
Ca	mmol/L	2.64	2.68	2.65	2.68	0.03	0.19
K	mmol/L	6.78	6.67	6.95	6.87	0.14	0.62

ALB, albumin; ALT, alanine aminotransferase; ALP, alkaline phosphatase; AMY, amylase; AST, aspartate aminotransferase; CHOL, cholesterol; CK, creatine kinase; GLOB, globulin; GLUC, glucose; Na, sodium; Mg, magnesium; Cl, chloride; Ca, calcium; K, potassium; P, phosphorus; SEM, standard error of mean; TBIL, total bilirubin; TP, total protein; U, units.

Values within the same row with different superscripts differ at *P* ≤ 0.05.

Elevation of serum enzymes alanine aminotransferase (ALT), aspartate aminotransferase (AST), serum alkaline phosphatase (ALP), and creatinine kinase (CK) in birds is associated with hepatopathy or muscle damage (Valchev et al., 2014). In the current study, there was no significant increase noticed in these enzymes in AP supplemented animals; they were within published reference values (Meluzzi et al., 1992). Glucose levels were consistent with Mabelebele et al. (2017) observations for clinically healthy broilers and were not different among evaluated groups.

In birds, circulating uric acid levels reflect protein catabolism in the body, and since they are excreted by kidney tubules, they are indirect indicators of kidney health (Huff et al., 1975). In this study, there were no statistical differences in the uric acid levels among TG and they were within the normal range (120 to 890 µmol/L) for healthy broiler birds. (Rezende et al., 2017).

Serum proteins are responsible for maintaining oncotic pressure within blood vessels and serve as indicators of liver health. Total protein (TP) and albumin (ALB) in the TG showed no statistically significant differences and were within the reference range of 25.8 to 52.2 g/L and 10.0 to 26.4 g/L, respectively (Meluzzi et al., 1992).

There were no statistically significant differences between control and TG for any of the biochemical parameters, except an increase in the level of phosphorus at the highest dose of AP. Serum phosphorus in 200,000 U/kg was elevated (*P* < 0.05) compared to control (2.44 vs. 2.29 mmol/L, respectively). However, these values are within the normal reference range of 2.0 to 2.7 mmol/L. There were four birds out of thirty in 200,000 U/kg with values marginally above reference maximum range (i.e., 2.8, 2.8, 2.8, and 2.9 mmol/L) which led to an overall increase in the serum phosphorus values. Thus, the difference in serum phosphorus is attributed to individual biological variation and has no clinical relevance.

None of the enzyme supplemented groups were statistically different from control for serum magnesium. A higher (*P* < 0.05) serum magnesium was found in the 200,000 U/kg compared to 20,000 U/kg (0.963 vs. 0.907, respectively). However, the increase in magnesium level was not dose-dependent and there was no clinical correlation with any other parameter. It is interpreted as not biologically relevant, and not adverse. Both values of 20,000 and 200,000 U/kg were well within control ranges 0.800 to 1.100 mmol/L, which is consistent with the observations made by Mabelebele et al. (2017).

An increase in cholesterol was observed in 200,000 U/kg compared to 20,000 U/kg. None of the enzyme supplemented groups were statistically different from control and all values were within the published range of serum cholesterol in broilers 3.23 to 5.17 mmol/L (Regar et al., 2019). These statistically significant variations had no clinical relevance and hence are attributed to individual biological variations. Overall, flock mortality was 0.8%, with no statistically significant differences among TG. Based on these results, it can be inferred that feeding this novel microbial-derived AP up to, and including, 200,000 U/kg of feed was extremely well tolerated by broiler chickens for a period of 42 d post-hatch.

In Study 2, blood samples from 20 randomly selected pigs (10 males and 10 females) were collected at the beginning of the study (day 0), to establish the baseline values, as per guidelines on the safety for porcine species (EFSA, 2017). On day 42 after weaning (end of study), blood samples were collected from one male and one female from each pen (*n* = 60 total). Results from statistical analysis of hematology, coagulation (Table 6), and clinical chemistry (Table 7) have been summarized.

**Table 6.** Hematological, differential cell count, and coagulation parameters after 42 d of in-feed supplementation with microbial-derived alkaline phosphatase in weaned pigs: Study 2

Item	Unit	Baseline	Alkaline Phosphatase, U/kg				SEM	*P*-value
			0	12,000	20,000	200,000		
*n*	- - -	20	12	16	16	16	- - -	- - -
RBC	×10^12^/L	6.81 ± 0.15	6.77	7.16	7.17	6.80	0.16	0.15
HB	g/L	118 ± 2	115	127	122	121	3	0.07
HCT	L/L	0.377 ± 0.007	0.358	0.398	0.387	0.376	0.010	0.07
MCV	fl	55.6 ± 0.9	53.3	55.7	53.9	55.5	1.0	0.26
MCH	pg.	17.5 ± 0.3	17.0	17.7	17.1	17.8	0.3	0.22
MCHC	g/L	315 ± 2	319	319	317	322	2	0.62
PLT	×10^9^/L	716 ± 30	539	478	433	448	38	0.28
WBC	×10^9^/L	14.8 ± 0.8	25.1^a^	18.9^a,b^	17.9^b^	17.7^b^	1.6	0.02
NEUT	×10^9^/L	7.41 ± 0.54	6.99	6.71	6.63	5.93	0.41	0.33
EOS	×10^9^/L	0.328 ± 0.036	0.256	0.310	0.277	0.184	0.035	0.08
BASO	×10^9^/L	0.084 ± 0.009	0.606^a^	0.222^a,b^	0.169^b^	0.178^b^	0.096	0.02
MONO	×10^9^/L	0.54 ± 0.04	1.09	0.84	0.82	0.70	0.10	0.10
LYMPH	×10^9^/L	6.42 ± 0.51	16.3^a^	10.6^b^	9.8^b^	10.5^b^	1.4	0.02
PT	sec.	13.3 ± 0.2	13.9	13.2	13.3	14.0	0.3	0.14
Fib	g/L	2.58 ± 0.09	1.76	1.96	1.81	1.84	0.11	0.60
APTT	sec.	20.8 ± 1.0	50.8	49.6	47.2	52.0	3.1	0.70

APTT, activated partial thromboplastin time; BASO, basophils; EOS, eosinophils; Fib, fibrinogen; HB, hemoglobin; HCT, hematocrit; LYMPH, lymphocytes; MCV, mean corpuscular volume; MCH, mean corpuscular hemoglobin; MCHC, mean corpuscular hemoglobin concentration; MONO, monocytes; NEUT, neutrophils; PT, prothrombin time; PLT, platelet count; RBC, red blood cells; SEM, standard error of mean; U, units; WBC, white blood cells.

Values within the same row with different superscripts differ at *P* ≤ 0.05.

**Table 7.** Clinical chemistry parameters after 42 d of in-feed supplementation with microbial-derived alkaline phosphatase in weaned pigs: Study 2

Item	Unit	Baseline	Alkaline Phosphatase, U/kg				SEM	*P*-value
			0	12,000	20,000	200,000		
*n*	- - -	20	12	16	16	16	- - -	- - -
AST	U/L	42.2 ± 2.6	39.5	42.0	43.5	42.9	3.5	0.87
ALT	U/L	40.4 ± 2.0	35.6	34.9	35.1	32.6	2.1	0.76
CK	U/L	580 ± 56	1,378	1,491	1,661	1,687	358	0.93
ALP	U/L	573 ± 36	297	347	301	328	15	0.08
BILES	µmol/L	13.2 ± 1.8	17.1	17.3	15.6	19.8	4.2	0.91
BUN	mmol/L	3.17 ± 0.17	4.61	5.14	4.64	4.35	0.23	0.12
CREAT	mg/dL	91.7 ± 3.5	62.6	66.8	62.3	66.8	2.4	0.37
TP	g/L	53.1 ± 0.7	56.8	56.3	56.3	55.2	1.0	0.67
BIL	µmol/L	6.59 ± 0.63	1.41^a,b^	1.33^b^	1.33^b^	3.02^a^	0.42	0.02
ALB	g/L	34.3 ± 0.8	31.6	31.7	32.3	31.8	0.9	0.94
GLOB	g/L	18.8 ± 0.8	24.7	24.6	24.5	23.4	0.8	0.61
CHOL	mmol/L	4.06 ± 0.41	1.66	1.44	1.64	1.77	0.09	0.09
AMY	U/L	1,424 ± 70	1,577	1,835	2,037	1,694	143	0.17
GLUC*	mmol/L	7.16 ± 0.26	5.98^a,b^	6.63^a^	6.33^a^	4.81^b^	0.29	0.0005
P	mmol/L	3.00 ± 0.07	3.36	3.53	3.46	3.49	0.09	0.64
Na	mmol/L	141 ± 1	141	143	141	142	1	0.31
Mg	mmol/L	1.09 ± 0.03	0.96	1.03	1.00	1.01	0.03	0.46
Cl	mmol/L	104 ± 1	101	102	101	101	1	0.66
Ca	mmol/L	2.65 ± 0.02	2.73^a,b^	2.78^a^	2.69^a,b^	2.61^b^	0.04	0.02
K	mmol/L	4.05 ± 0.08	5.48	5.64	5.41	5.66	0.28	0.89

AMY, amylase; AST, aspartate aminotransferase; ALB, albumin; ALT, alanine aminotransferase; ALP, alkaline phosphatase; BIL, bilirubin; BUN, blood urea nitrogen; CHOL, cholesterol; CK, creatine kinase; CREAT, creatinine; GLOB, globulin; GLUC, glucose; PHOS, phosphorus; Na, sodium; Mg, magnesium; Cl, chloride; Ca, calcium; K, potassium; SEM, standard error of mean; TP, total protein; U, units.

The Wald test for pen as random effect for glucose resulted in a variable ratio of 1.50 ± 0.18 (S.E.), which is different from zero at *P* = 0.008. This means that a significant random effect of pen was detected for blood glucose.

Values within the same row with different superscripts differ at *P* ≤ 0.05.

Hematological indices showed no significant findings associated with red blood cells (RBC), hematocrit (HTC), mean corpuscular volume (MCV), mean corpuscular hemoglobin (HB), mean corpuscular hemoglobin concentration (MCHC), and platelets (PLT). Hemoglobin tended (*P* = 0.07) to be higher in 12,000 U/kg when compared with control (127 vs. 115 g/L, respectively). The values were within published porcine reference range (Cooper et al., 2014; Ventrella et al., 2017). There was no correlation with the other red cell parameters suggesting a lack of biological significance. The changes were ascribed to individual biological variation.

White blood cells count was lower (*P* = 0.02) in 20,000 U/kg and 200,000 U/kg compared to control group (17.9 and 17.7 vs. 25.1 × 10^9^/L, respectively). This largely reflected a high degree of variation at the time of venipuncture, likely due to physiological leukocytosis. A transient leukocytosis occurs in response to physical exercise or due to increased levels of adrenalin in healthy individuals (Iversen, 1992). The two highest individual values were in the control group (45.64 and 52.63 × 10^9^/L). In both instances, this leukocytosis was due to a lymphocytosis (37.52 and 42.52 × 10^9^/L), which was higher than any TG (16.3 vs. 10.6, 9.8, and 10.5 × 10^9^/L for control; 12,000; 20,000; and 200,000 U/kg, respectively; *P* = 0.02). The leukocyte counts in 12,000; 20,000; and 200,000 U/kg were within the published reference range for pigs 15.6 to 38.9 × 10^9^/L (Klem et al., 2010; Cooper et al., 2014) indicating a lack of biological significance. Basophil counts were lower (*P* = 0.02) in 20,000 and 200,000 U/kg when compared with controls; however, in general, variations in basophil numbers are of questionable significance.

There were no statistically significant differences between control and TG for the biochemical parameters of AST, ALT, CK, ALP, bile acids, blood urea nitrogen (BUN), creatinine, total protein, albumin, globulins, cholesterol, amylase, phosphate, sodium, magnesium, chloride, and potassium. The results were within the normal range previously described by Cooper et al. (2014).

Bilirubin was lower (*P* = 0.02) in 12,000 and 20,000 U/kg when compared with 200,000 U/kg. There is no clinical relevance to decreased bilirubin levels. There were no indicators of hepatic compromise (Kaneko et al., 1997).

Blood glucose was higher (*P* = 0.0005) in 12,000 and 20,000 U/kg when compared with 200,000 U/kg. Most importantly, none of the AP supplemented TG differed from control animals or exceeded the maximum normal range of control animals (4.52 to 8.48 mmol/L); all values were within the reference range published earlier 5.1 to 8.3 mmol/L (Egeli et al., 1998). Blood glucose is highly labile, and the adrenergic response associated with handling and venipuncture is a common cause of spurious hyperglycemia (Imlan et al., 2020). Further, the Wald test for pen as random effect for glucose resulted in a variable ratio of 1.50 ± 0.18 (SE), which is different from zero at *P* = 0.008. This indicates a random pen effect with relation to blood glucose. Thus, variations in glucose levels are most likely attributed to blood sampling sequence, fed status (animals were not fasted prior to blood collection), and/or animal stress and not related to in-feed supplementation of AP.

Calcium was lower (*P* = 0.02) in 200,000 U/kg compared to 12,000 U/kg (2.61 vs. 2.78 mmol/L, respectively). None of the AP supplemented TG differed from control animals or exceeded the maximum normal range of control animals. However, due to lack of dose-dependency, the result cannot be attributed as a true treatment effect. There was no biological or clinical correlation to varying calcium levels, indicating a lack of biological significance.

Treatment with AP in-feed was well tolerated in pigs according to results from hematology, coagulation, and biochemical parameter assessments when included at 12,000; 20,000; and 200,000 U/kg of feed.

### Intestinal Permeability

Maintaining gastrointestinal health in production animals is essential to achieve maximum production potential, through improved feed conversion ratio. Multiple components of the gut such as GALT, gut mucosa, Paneth cells, endocrine cells, absorptive enterocytes, and tight junctions between intestinal enterocytes play a crucial role in gut health and act as the first line of defense against pathogens. The brush-border enzyme, IAP, is a part of the gut mucosal defense system that prevents bacterial invasion by dephosphorylating LPS which is a component of the bacterial cell wall of Gram-negative bacteria in the gut (Melo et al., 2016) and other potentially pro-inflammatory phosphate monoesters. Compromised intestinal health leads to intestinal microbiome shift (dysbiosis), increased intestinal permeability or leakage, leading to systemic inflammation and poor nutrient absorption.

**Figure 1.**
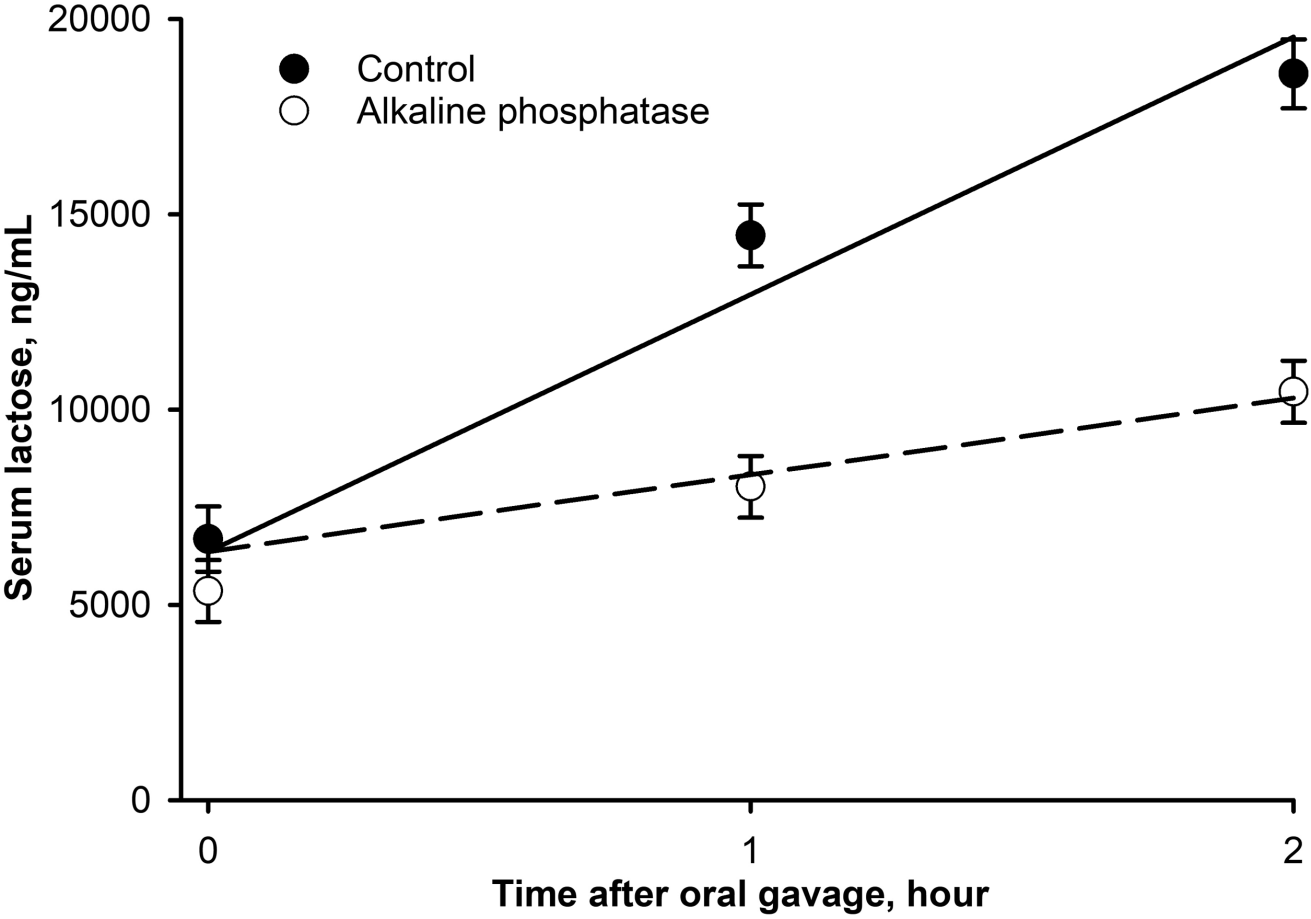
Slope-ratio comparison of serum lactose after an oral gavage with lactose in broiler chickens supplemented in-feed with microbial-derived alkaline phosphatase (AP) for 28 d: Study 3. The common intercept was 6,354.5 with a slope of 6,595.1 for control and 1,975.9 for AP, *R*^2^ = 0.766, slopes differ at *P* = 0.0001. Relative paracellular permeability was determined to be 100% for control birds and 29.96% for AP-supplemented birds.

Study 3 was designed to assess the effect of in-feed AP supplementation on intestinal permeability and pigment absorption. Birds were fed experimental diets supplemented with 0 or 10,000 U of AP per kg of feed for 28 d. Intestinal permeability was quantified after the administration of a single oral gavage dose of lactose, which is an indigestible disaccharide for birds due to the lack of the enzyme lactase in their gastrointestinal tract (Hamilton and Mitchell, 1924; Chotinsky et al., 2001; Mozdziak et al., 2003). Therefore, the appearance of lactose in blood after oral gavage is interpreted as a direct measurement of intestinal permeability. In this study, supplementation of AP reduced paracellular permeability by 70% in vivo as indicated by decreased (*P* = 0.0001) slope for serum lactose appearance in supplemented birds compared with controls as shown in Figure 1. This finding suggests an improvement in intestinal tight junction structure and/or function when AP was incorporated into the diet of broiler chickens.

Skin pigmentation values with and without AP supplementation are shown in Table 8. Dietary pigment uptake has been suggested as an indirect measurement of intestinal integrity. Improved xanthophyll absorption from the intestine leads to increased yellow pigmentation of the skin in broilers (Frade-Negrete et al., 2016). Yellow skin pigmentation is an economically important trait in many countries like Mexico, Guatemala, Spain, Italy, and others. In the current study, skin pigmentation values were not different between TG at day 14 of study, which was just prior to the introduction of xanthophyll pigments in the diet. After 14 d of in-feed AP supplementation, skin yellowness (i.e., b* value) value was increased (*P* < 0.0001) by 7.3% compared to control birds. The combined results of intestinal permeability and skin pigmentation can be interpreted to suggest that oral supplementation of AP improves gut health and perhaps nutrient absorption because skin pigment deposition is correlated with fat digestion and absorption (Hernández-Velasco, et al., 2014 and Gilani, 2017).

**Table 8.** Skin pigmentation values after 28 d of in-feed supplementation with microbial-derived alkaline phosphatase in broiler chickens: Study 3

Item	Alkaline Phosphatase, U/kg		SEM	*P*-value
	0	10,000		
Day 14				
L*	60.74	60.67	0.31	0.88
a*	8.49	8.77	0.18	0.29
b*	6.31	6.20	0.27	0.77
Day 28				
L*	59.57	59.33	0.17	0.33
a*	8.00	8.16	0.14	0.41
b*	17.36	18.63	0.21	<0.0001

L* values range from black (0) to white (100); a* values range from red (+) to green (-); and b* values range from yellow (+) to blue (-); SEM, standard error of mean.

## CONCLUSION

It can be concluded that microbial AP produced by *P. lentus* strain CMG3709 as a feed additive is safe for broiler chickens and weaned pigs at the recommended level of 12,000 U per kg of feed and has a wide margin of safety. The survival rate in all the three studies was more than 98%, with no biologically significant differences among treatment groups, and the overall health of the animals was good. In fact, supplementation of microbial AP positively impacted gut health. This conclusion can be extended to birds reared for laying, other poultry species and pigs reared for fattening, and other swine species.
